# Effectiveness of vitamin D2 compared with vitamin D3 replacement therapy in a primary healthcare setting: a retrospective cohort study

**DOI:** 10.5339/qmj.2022.35

**Published:** 2022-08-04

**Authors:** Esmail Mohammad Alayed Albarri, Ahmed Sameer Alnuaimi, Doaa Abdelghani

**Affiliations:** ^1^Al Wajbah Health Center, Primary Health Care Corporation, Qatar E-mail: esmilbarri@yahoo.com; ^2^Clinical Affairs - Clinical Research, Primary Health Care Corporation, Qatar

**Keywords:** primary, health, care,, vitamin, D, deficiency,, vitamin, D2, (ergocalciferol),, and, vitamin, D3, (cholecalciferol)

## Abstract

Introduction: Vitamin D deficiency is a worldwide public health concern, which can lead to severe diseases, such as rickets in children and osteomalacia in adults. Most studies have compared equimolar unit-to-unit doses of vitamin D2 and D3.

Objectives: The current study aimed to answer the research question: “How effective is vitamin D2 (600,000 U/1.5 ml) compared to vitamin D3 (300,000 U/1 ml) parenteral supplementation for raising serum vitamin D levels in adult patients treated in a primary health care setting?”

Setting: Primary Health Care Corporation (PHCC) runs 28 health centers distributed throughout the State of Qatar and its capital city, Doha. Qatar is on the east coast of the Arabic peninsula, with very hot and sunny summers and a desert climate.

Study design: This was a retrospective observational cohort study.

Method: A total of 15,716 participants were recruited following ethical approval. They were identified by electronic medical records (EMR) describing the clinical encounters of individuals aged 18 to 60-years-old who attended a health center operated by the PHCC during the 3.5-year study period from January 1, 2017 to June 30, 2020. The PHCC EMR system uses SNOMED codes (a systematically organized computer-processable collection of medical terms providing codes, names, synonyms, and definitions implemented for clinical documentation and reporting). Four study groups were created depending on the type of vitamin D injection and the oral form of replacement therapy. The analysis scheme used the serum vitamin D level within the preceding 4 weeks (pretreatment), followed by administration of the treatment dose. The post-treatment serum testing value should have been available within a maximum of 12 weeks. The Statistical Package for Social Sciences (IBMSPSS; IBM Corp., Armonk, NY, USA) version 23 software was used for the statistical analysis.

Results: Four treatment options were compared, including a vitamin D2 injection, a vitamin D3 injection, combined use of a vitamin D2 injection + a D2 tablet, and combined use of a vitamin D3 injection + a D2 tablet. All four treatment groups were associated with a statistically significant increase in serum vitamin D within a maximum of 12 weeks of follow-up. The vitamin D2 injection alone was associated with the lowest increase in serum concentration by a mean of 3.2 ng/ml. In contrast, the vitamin D3 injection alone or with a D2 tablet increased serum vitamin D by 6.1 and 5.6 ng/ml, respectively. Using the combination of a vitamin D2 injection and a tablet only added a marginal increase of 2.3 ng/ml in serum vitamin D on top of the 3.2 ng/ml increase attained after administering the D2 injection alone.

Conclusion: Utilizing vitamin D3 in an injectable form is the best choice to restore severe vitamin D deficiency. Furthermore, it was superior to the injectable form of vitamin D2, even though vitamin D2 has double the molar units.

## Introduction

Vitamin D is a fat-soluble vitamin consisting of vitamin D2 (ergocalciferol) and vitamin D3 (cholecalciferol). The primary source of vitamin D2 is plants, and D2 can be manufactured synthetically, whereas vitamin D3 is synthesized in the human skin from 7-dehydrocholesterol after exposure to the sun. Both forms of vitamin D are inactive until processed by enzymatic hydroxylation^1^. Vitamin D is involved in the function of vital organs, such as the kidneys, intestinal mucosa, and bones to regulate calcium and phosphate metabolism. In addition, severely low levels can lead to rickets in children and osteomalacia in adults^2^.

Vitamin D is also essential in calcium and phosphorous homeostasis and is associated with parathyroid hormone. A vitamin D deficiency is thought to be associated with osteoporotic and stress-related fractures^3^. Some studies have linked vitamin D deficiency to colon cancer, arthritis, diabetes mellitus, and cardiovascular diseases^4,5,6,7^. Vitamin D deficiency is considered a significant healthcare concern worldwide in all age groups. It is more of a problem for residents in countries located at high latitudes where ultraviolet radiation is insufficient. In addition, residents of developed countries suffer despite the fortification of vitamin D in their food for many years^8^. A recent study in Qatar showed that the prevalence of severe vitamin D deficiency (serum level < 10 ng/ml) among adults attending the Primary Health Care Corporation (PHCC) health centers and aged 18–65 years was 14%. Using less stringent criteria for defining vitamin D deficiency (serum level < 20 ng/mL) increases the prevalence rate to 71.4%^9^.

Many factors are involved in the etiology of vitamin D deficiency, including lack of sun exposure and insufficient consumption of foods rich in vitamin D^10^. Other factors associated with low serum vitamin D levels are age, gender, clothing style, darker skin, socioeconomic status, and body mass index^9^. Moreover, the lifestyle in the Arabian Gulf area is another major factor in vitamin D deficiency because many people travel in their cars rather than walk, run, or cycle because of convenience and the hot climate^6,11,12^.

The preferred marker of vitamin D status is 25(OH)D because it is the principal circulating form of vitamin D in the blood with a half-life of 2–3 weeks^13^. Recent evidence suggests that inter-laboratory variability in 25(OH)D assays could provide an unclear interpretation of low vitamin D serum levels^14^.

The ideal serum 25(OH)D concentration is controversial. The majority of classifications consider that a severe vitamin D deficiency is defined as < 12 ng/ml (30 nmol/L) 25(OH)D. Moderate deficiency is defined as 12–20 ng/ml (30–50 nmol/L) 25(OH)D. Mild deficiency is defined as 25(OH)D >20 ng/ml (50 nmol/L)^15^. PHCC in Qatar considers a severe deficiency to be < 10 ng/ml and a moderate deficiency as < 20 ng/ml.

Recent clinical practice recommendations consider that vitamin D2 and vitamin D3 have the same equivalence in the therapeutic field^2^. However, numerous recent clinical trials have evaluated the ability of equimolar dosing regimens of vitamin D2 vs. vitamin D3 by their capability to increase and sustain the serum total 25(OH)D level^16^. Although most studies have found that orally-administered vitamin D3 increases total serum 25D more vigorously than D2^17,18,19,20^, others have found them to have the same efficacy^21,22^. In contrast, studies that show greater efficacy for vitamin D3 may be limited by small sample size^23^. Furthermore, most studies compared the equal molar quantity of either vitamin D2 or vitamin D3 to elevate serum 25(OH)D.

The current study aimed to answer the research question: “How effective is parenteral supplementation of vitamin D2 (600,000 U/1.5 ml) compared to vitamin D3 (300,000 U/1 ml) on serum vitamin D levels in adult patients treated in a primary healthcare setting?”

## Methods

*Study settings:* The PHCC is a publicly funded primary healthcare provider in Qatar. It provides healthcare services to a large part of the country's population and runs 27 health centers distributed throughout the country^24^. PHCC operates an EMR system called CERNER, which was introduced in July 2016. Qatari citizens and ex-pats who live with their families in Qatar can access and utilize healthcare services provided by the PHCC after registering and paying a nominal annual fee.

*Study design:* This was a retrospective observational cohort study.

*Study population:* The data analyzed covers the EMR describing the clinical encounters of individuals aged 18–60-years-old who presented at a health center operated by the PHCC during the 3.5-year study period from January 1, 2017 to June 30, 2020. The inclusion criterion for study participants was having a prescription for at least one dose of a vitamin D2 (600,000 units) or D3 (300,000 units) injection during the study period.

*Study variables:* The following variables were extracted from the EMR for the targeted study population:

• All serum vitamin D measurements and their dates.
• All prescribed vitamin D2 (600,000 units) or vitamin D3 (300,000 units) injections and tablets with the dates administered.
• Sociodemographic variables, including age at first serum vitamin D measurement (before starting vitamin D replacement therapy), gender, and nationality.
• SNOMED codes for comorbidities including diabetes mellitus, dyslipidemia, asthma, chronic obstructive pulmonary disease, hypertension, cerebrovascular disorders, and chronic kidney disease^25^.

*Data Collection:* The PHCC EMR system uses SNOMED codes (a systematically organized collection of medical terms providing codes, names, synonyms, and definitions used in clinical documentation and reporting). SNOMED International is a not-for-profit organization that owns and maintains this medical coding system. These codes are quality-controlled and reviewed by the Business Health Intelligence (BHI) department of PHCC. The BHI department is responsible for translating SNOMED codes into ICD-10 codes (International Classification of Disease, Tenth Revision) and continuously updates the coding manual at monthly intervals with new codes used in the organizational database^26^. The BHI department provided a complete list of variables for the study population using custom-made filters.

*Data cleaning:* A total of 21,269 adults had their first vitamin D injection (D2 or D3) during the study period. Among them, 785 had their first serum vitamin D test after being prescribed the treatment with no available laboratory test before the prescription. These individuals were excluded from the analysis. Another 4,768 individuals had a recorded value for serum vitamin D older than 4 weeks before the first vitamin D injection. This group of study participants was also excluded from the analysis.

The database was restructured to allow a retrospective cohort study analysis using the study date range, with each participant's interaction with the health care system. The aim was to create a paired data design in the form of a pretreatment and post-treatment comparison of serum vitamin D measurements. Four study groups were created depending on the type of vitamin D injection and the oral form of replacement therapy that they may have received as an add on. The primary unit of analysis was the treatment intervention. To qualify for inclusion in the database, a participant should be preceded by a serum vitamin D test measurement within the previous 4 weeks (pretreatment), followed by administration of the treatment. The post-treatment serum testing value should be available within a maximum of 12 weeks.

*Data analysis:* IBMSPSS version 23 software (SPSS Inc., Chicago, IL, USA) was used for the statistical analysis. A P value < 0.05 was considered significant. The paired *t*-test was used to assess the statistical significance of mean change in a quantitative normally distributed variable (serum vitamin D) after treatment. Cohen's d was used to assess the magnitude of the effect size of each treatment option compared to the vitamin D2 injection alone. A multiple linear regression model was employed to measure the net and independent effect of the treatment options on the magnitude of change in serum vitamin D attributed to the treatment after controlling for possible confounding effects of age, gender, and comorbidities.

Vitamin D deficiency status was measured at two pre-identified cut-off values. The severe deficiency status was defined as a serum vitamin D concentration < 10 ng/ml. Less stringent criteria for defining deficiency status was the < 20 ng/ml cut-off value. The effect of the four treatment options in overcoming the deficiency status after treatment was measured by the paired odds ratio^27^.

*Quality control measures:* An extensive review of the literature was undertaken during the preparative phase of the study. The authors are responsible for data collection in collaboration with the BHI department.

*Ethical considerations:* The study presented minimal risk of harm to the participants. The original research proposal was an anonymized data request. This research proposal was approved by the Research Sub-Committee of the PHCC (PHCC/DCR/2020/01/006). This study was conducted according to generally accepted ethical principles.

## Results

**Figure 1.**
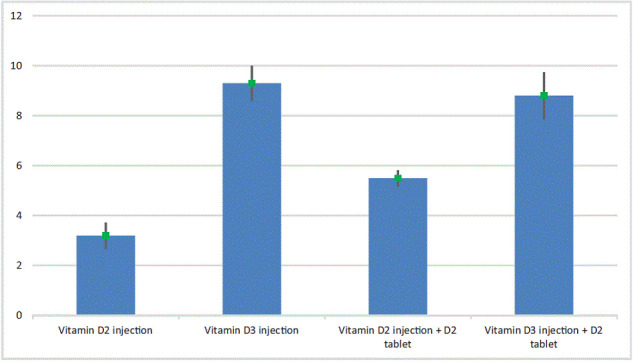
Mean change in serum vitamin D (ng/ml) after treatment.

As shown in Table 1 and Figure 1, all four treatment groups were associated with a statistically significant increase in serum vitamin D within a maximum of 12 weeks of follow-up. A vitamin D2 injection alone was associated with the smallest increase in serum concentration by a mean of 3.2 ng/ml. In contrast, the vitamin D3 injection alone or with a D2 tablet conferred an extra 6.1 and 5.6 ng/ml of serum vitamin D, respectively, on top of the changes introduced by the D2 injection alone. This additional effect of the vitamin D3 injection compared to the D2 injection was classified as a strong effect (Cohen's d >0.6). Using a combination of a vitamin D2 injection and a tablet only conferred a marginal increase of 2.3 ng/ml in serum vitamin D on top of the 3.2 ng/ml increase attainable with an injection alone.

The effect of age, gender, and six comorbidities on the magnitude of change in serum vitamin D in response to replacement therapy was evaluated in Table 2. Almost all of the explanatory variables had a statistically significant but weak effect (Cohen's d < 0.3) on the treatment response measured by the change in serum vitamin D. In addition, age and chronic kidney disease had no significant effect on the magnitude of the response. Male patients had a better response to replacement therapy than females. Comorbidities were associated with a smaller magnitude of response in the serum vitamin D level to replacement therapy.

A multiple linear regression model was used to assess the net and independent effects of the explanatory variables on changes in serum vitamin D after treatment. A conclusion similar to the bivariate model considered in Table 2 was reached here. The vitamin D3 injection alone or with a D2 tablet was associated with the highest treatment response compared to a vitamin D2 injection alone after adjusting for age, gender, and comorbidities. The model was statistically significant, as illustrated in Table 3.

The effectiveness of the four types of treatments in reducing the prevalence of vitamin D deficiency at the two preset cut-off values was assessed in Tables 4 to 15. A serum vitamin D concentration of < 10 ng/ml was identified as a severe vitamin D deficiency, while < 20 ng/ml was identified as a vitamin D deficiency. In addition, the magnitude of the response to treatment was measured by the paired odds ratio. This ratio measures the probability of achieving the treatment target (overcoming deficiency status after treatment) compared to developing a new deficiency status after treatment (treatment failure). As a result, the beneficial treatment effect was higher and more pronounced when the target for the four treatment types was to correct a severe deficiency as opposed to a deficiency status. In addition, a vitamin D3 injection, whether alone or combined with a D2 tablet, provided the most substantial treatment effect.

## Discussion

Clinicians must often reconcile the encouraging results of well-designed studies that have evaluated treatment effectiveness against the apparent replication in everyday medical practice on ambulatory patients in a primary care setting. Therefore, this retrospective cohort study was designed to compare the effectiveness of two forms of vitamin D parenteral supplementation available in the primary healthcare setting, such as vitamin D2 (600,000 U/1.5 ml) and vitamin D3 (300,000 U/1 ml), on improving serum vitamin D levels in adult patients. In addition, the treatment effect of parenteral supplementation in the current study was adjusted for the confounding effect of oral supplementation forms readily available in the health center. Four treatment options were compared. These included a vitamin D2 injection, a vitamin D3 injection, combined use of a vitamin D2 injection + a D2 tablet, and a vitamin D3 injection + a D2 tablet. The results showed that both vitamin D treatments (injectable forms of vitamin D2 and vitamin D3) increased serum vitamin D levels. This finding is consistent with many studies indicating that both types of vitamin D replacement therapy (D2 and D3) increase serum 25(OH)D levels using various vitamin D concentrations and dosing schedules^28,29,30,31,32,33^. In contrast, few studies failed to determine a significant effect of both vitamin D2 and vitamin D3 treatment in their participants. These studies used a small sample size ( < 100 participants in both studies and low doses of vitamin D2 and D3 (1,000 IU daily)^22,21^.

A small sample-sized study compared the treatment response of 50 participants to a bolus dose of 300,000 IU IM (injectable) vitamin D2 (ergocalciferol) to that of 19 participants who received 300,000 IU oral vitamin D3 (cholecalciferol). The authors concluded that both preparations were practical, well-tolerated, and safe. Vitamin D3 had greater potency than equimolar vitamin D2^34^. To evaluate this recommendation, the participants in our study received a double concentration of vitamin D2 compared to D3 (600,000 IU D2 compared to 300,000 IU D3). Nevertheless, vitamin D3 replacement therapy was superior to that of D2 in achieving higher serum vitamin D levels, despite the double concentration of vitamin D2.

The current study demonstrated that a vitamin D3 injection alone or combined with a D2 tablet achieved the best treatment response compared to a vitamin D2 injection or a vitamin D2 injection + a D2 tablet in the primary care setting. This finding agrees with a systematic review and meta-analysis concluding that vitamin D3 replacement is more effective than vitamin D2^35^ However, that meta-analysis was the subject of controversy and considerable criticism because of the small number of studies included and the inadequate sample sizes of the participants in each of the studies.

Another observation made by the current study was the large difference in treatment effect between the vitamin D3 injection and the vitamin D2 injection. The former is twice as effective as D2 when used alone and three times more effective when combined with an oral tablet. A similar conclusion was reached in another study comparing 50,000 IU oral administration of vitamin D2 with D3, which showed an 87% increase in D3 potency compared to D2^36^. Another study revealed that vitamin D2 replacement therapy is almost one-third the potency of vitamin D3^37^.

The current study showed that combining the less potent form of vitamin D in parenteral replacement therapy (vitamin D2) with oral tablets resulted in a doubling of the potency of the injection alone. This result could be explained by the time needed to reach the steady-state concentration. The half-life of these medications ranges from 5 weeks to 5 months^38^, and the vitamin D binding protein affinity of the injection and tablet, as well as the oily nature of injectable therapy, are other reasons. In addition, combining parenteral vitamin D3 with a D2 tablet was more effective than the isolated use of the parenteral preparation for managing a severe vitamin D deficiency. However, this added advantage of vitamin D2 tablets failed to show when the absolute mean change in serum vitamin D was examined. This may reflect the varying focus of the managing physician or the individual (whenever possible) in achieving higher serum levels Vs simply achieving the target of overcoming the deficiency status.

Including a large number of individuals with comorbidities in this study enabled an in-depth analysis of the treatment effect. In general, having comorbidities (diabetes mellitus, hypertension, dyslipidemia, cerebrovascular disorder, asthma, or chronic obstructive pulmonary disease) was associated with a significant but weak effect (Cohen's d < 0.3) on the treatment response based on changes in serum vitamin D for both treatments. This may relate to the multiple medications that these patients may be taking to control their conditions. The only exception was chronic kidney disease, which had no statistically significant effect on the magnitude of the response.

The current study demonstrated that the age of the patients was not associated with a significant difference in vitamin D levels post-treatment for any type of regimen. Moreover, male participants had a significantly better response than females. This difference could be related to many factors, such as the dietary habits of the participants or the amount of sun exposure, which is influenced by the exposed body surface. In general, social customs in the Middle East prohibit females from openly exposing most parts of their body.

Another question that was addressed by the current study was “is it better to target moderate or severe vitamin D deficiency with replacement therapy?” Targeting severe deficiency was associated with a much better treatment response compared to treating a moderate deficiency. This outcome was evident when using vitamin D3 alone or with a D2 tablet and the same was applied to using the vitamin D2 injection alone or with a vitamin D2 tablet. This outcome has financial implications, considering the cost of medication used to restore normal levels of vitamin D and the cost of hospitalization due to a vitamin D deficiency. It has been suggested that of the 30 leading causes of death in the United States in 2010, 19 were linked to low vitamin D status, including various forms of cardiovascular disease, various cancers, diabetes mellitus, Alzheimer's disease, falls, and fractures in the elderly^39^. If the population of the United States was to increase their vitamin D status to 40 ng/mL, we could expect to see a potential reduction of as much as 336,000 deaths each year (out of 2.1 million deaths attributed to the diseases of concern), so raising 25(OH)D concentrations would be a cost-effective route to reduce the burden of disease and increase life expectancy in the United States^40^.

In summary, using a vitamin D3 injectable form to correct a severe vitamin D deficiency is an appropriate choice to restore acceptable levels and has superiority over a vitamin D2 injectable form. The double molar units used to achieve the greater impact of vitamin D2 were insufficient to achieve a comparable improvement level to that with vitamin D3. The synergistic effect of a vitamin D2 tablet with a vitamin D2 injection is notable and could help to achieve a better treatment response.

## Limitations

The main limitation of this study arises from the fact that it was based on the patients’ electronic health records. This resulted in the inability to adjust for the confounding effect of vitamin D intake from food sources, sun exposure, smoking history, alcohol intake, and physical exercise status of the participants^13^. Another possible source of bias was the variable follow-up period, ranging from 1 to 12 weeks. In addition, the long-term outcomes of the intervention were not evaluated.

The number of injections received during the 3-month follow-up period would affect the resulting serum vitamin D level after treatment. However, this bias would have a small effect when comparing the two treatments (since it is a non-differential bias) as it affected both the same. In addition, the magnitude of the response was not the primary objective of this study, but rather the difference incurred by treatment choices in a primary health care setting.

## Conclusion

Vitamin D2 and vitamin D3 increased serum vitamin D levels, but doubling the vitamin D2 dose failed to match the better treatment response of vitamin D3. Relying on a vitamin D3 injectable form would be a preferable choice for treating the severe form of vitamin D deficiency in a primary care setting.

The vitamin D3 injectable form had a more favorable effect in terms of treating vitamin D deficiency and could be a more cost-effective option to achieve the targeted goal of treatment.

It would be interesting to undertake further investigations to compare the effectiveness of vitamin D2 and D3 in a randomized controlled trial (intervention compared to a control group).

### Acknowledgments

We gratefully acknowledge Dr. Ahmed Sameer Alnuaimi for his continuous guidance and assistance. Many thanks to the team at Al Wajbah Health Center, including Dr. Meshal Al Mesaifri (Health Center Manager), Dr. Alia Al Ruwaili (Deputy Health Center Manager), Dr. Muhammad Arsalan Zamir (Physician Lead), and the Pharmacy team.

Special thanks also to the Business Intelligence Department at the PHCC for their support in data extraction.

## Figures and Tables

**Table 1:** Mean change in serum vitamin D after four types of replacement therapies.

	Serum vitamin D ng/ml-Changes (after the first dose)					Difference in mean compared to the reference	Effect size (Cohen's d) compared to the reference	*P* value for difference in mean compared to the reference

	Range	Mean	SD	SE	N	category	category	category

Type of vitamin D prescribed								

vitamin D2 injection	(-16 to 50)	3.2	0.51	0.33	411	Reference		

vitamin D3 injection	(-17 to 48)	9.3	0.69	0.49	454	6.1	0.69	< 0.001

vitamin D2 injection + D2 tablet	(-18 to 47)	5.5	0.32	0.18	1585	2.3	0.32	< 0.001

vitamin D3 injection + D2 tablet	(-18 to 47)	8.8	0.94	0.27	997	5.6	0.94	< 0.001


P (ANOVA) <  0.001

P (Paired *t*-test) for each of the four treatments < 0.001

**Table 2:** Mean change in serum vitamin D after treatment with selected explanatory variables

	Serum vitamin D ng/ml-Changes (after the first dose)					Effect size (Cohen's d) compared to the reference	Difference in mean compared to the reference	*P* value for difference in mean compared to the reference

	Range	Mean	SD	SE	N	category	category	category

Gender								

Female	( − 17 to 50)	6.5	7.9	0.16	2501		Reference	

Male	( − 18 to 48)	7.2	9.4	0.31	946	0.08	0.7	0.0497

Age group (years)								

< 30	( − 10 to 47)	7	7.7	0.23	1119		Reference	

30–39	( − 18 to 50)	6.6	8.3	0.24	1224	− 0.05	− 0.4	1[NS]

40–49	( − 18 to 48)	6.5	8.8	0.32	754	− 0.06	− 0.5	1[NS]

50–60	( − 15 to 45)	6.4	9.7	0.52	350	− 0.06	− 0.6	1[NS]

History of Diabetes Mellitus								

Negative	( − 18 to 50)	6.9	8.4	0.16	2798		Reference	

Positive	( − 15 to 48)	5.6	8.1	0.32	649	− 0.16	− 1.3	< 0.001

History of Hypertension								

Negative	( − 18 to 50)	6.8	8.4	0.15	3021		Reference	

Positive	( − 15 to 39)	5.5	7.8	0.38	426	− 0.16	− 1.3	0.001

History of Dyslipidemia								

Negative	( − 18 to 50)	7	8.4	0.16	2727		Reference	

Positive	( − 15 to 48)	5.5	8.2	0.3	720	− 0.18	− 1.5	< 0.001

History of Cerebrovascular Disorder								

Negative	( − 18 to 50)	6.7	8.4	0.14	3394		Reference	

Positive	( − 9 to 36)	4.8	7.9	1.09	53	− 0.23	− 1.9	0.09[NS]

History of Chronic Kidney Disease								

Negative	( − 18 to 50)	6.7	8.3	0.14	3422		Reference	

Positive	( − 8 to 31)	6.8	9.4	1.88	25	0.01	0.1	0.97[NS]

History of Asthma / Chronic Obstructive Pulmonary Disease								

Negative	( − 18 to 50)	6.8	8.4	0.15	3037		Reference	

Positive	( − 15 to 48)	5.6	8.1	0.4	410	− 0.14	− 1.2	0.004


**Table 3:** Multiple linear regression model and the changes in serum vitamin D after treatment as the dependent (outcome) variable and the treatment type, gender, age, and history of selected chronic conditions as the explanatory variables.

	Unstandardized regression Coefficient (B)			

	estimate	95% confidence interval	P	Standardized Coefficients

(Constant)	3.5	(2.6 to 4.5)	< 0.001	

vitamin D3 (Cholecalciferol) injection compared to vitamin D2 injection	6.2	(5.2 to 7.3)	< 0.001	0.25

Combined (Cholecalciferol) injection [D3] + D2 tablet) compared to vitamin D2 injection	5.7	(4.8 to 6.6)	< 0.001	0.31

Combined (Ergocalciferol) injection [D2] + D2 tablet) compared to vitamin D2 injection	2.4	(1.5 to 3.2)	< 0.001	0.14

Male gender compared to female	1.3	(0.6 to 1.9)	< 0.001	0.07

History of Diabetes Mellitus	− 1.3	( − 2.1 to − 0.5)	< 0.001	− 0.06

History of Dyslipidemia	− 1.5	( − 2.2 to -0.7)	< 0.001	− 0.07

History of Asthma / Chronic Obstructive Pulmonary Disease	− 0.9	( − 1.7 to − 0.1)	0.036	− 0.03

History of Hypertension	− 0.7	( − 1.6 to 0.2)	0.13[NS]	− 0.03

History of Cerebrovascular Disorder	− 1.2	( − 3.4 to 1.1)	0.31[NS]	− 0.02

History of Chronic Kidney Disease	1.7	( − 1.5 to 4.9)	0.29[NS]	0.02

Age group (years)	− 0.01	( − 0.3 to 0.3)	0.98[NS]	− 0.001


P (model) <  0.001

Determination coefficient (R^2^) = 0.076

**Table 4:** Relative frequency (prevalence) of two definitions of serum vitamin D deficiency before and after treatment with a vitamin D2 injection.

vitamin D2 injection (Total examined	Before treatment		95% Confidence	After treatment		95% Confidence

N = 411)	N	%	Interval	N	%	Interval

Severe vitamin D deficiency (serum conc < 10 ng/ml)	124	30.2	(25.9 to 34.7)	41	10	(7.4 to 13.2)

vitamin D deficiency (serum conc < 20 ng/ml)	364	88.6	(85.2 to 91.4)	326	79.3	(75.2 to 83)


**Table 5:** Risk (paired odds ratio) of responding to vitamin D2 injection replacement therapy considering a severe serum vitamin D deficiency as the outcome compared to pretreatment deficiency status.

	Sever vitamin D deficiency (serum conc < 10 ng/ml)-After treatment (within 12 weeks)			Paired	95% Confidence Interval	

vitamin D2 injection	Negative	Positive	Total	OR	OR	P

Severe vitamin D deficiency (serum conc < 10 ng/ml)-Baseline (before treatment)						

Negative	280	7	287	13	(6 to 28)	< 0.001

Positive	90	34	124			

Total	370	41	411			


**Table 6:** Risk (paired odds ratio) of responding to vitamin D2 injection replacement therapy considering vitamin D deficiency as the outcome compared to pretreatment deficiency status.

	vitamin D deficiency (serum conc < 20 ng/ml)-After treatment (within 12 weeks)			Paired	95% Confidence Interval	

vitamin D2 injection	Negative	Positive	Total	OR	OR	P

vitamin D deficiency (serum conc < 20 ng/ml)-Baseline (before treatment)						

Negative	25	22	47	3	(2 to 5)	< 0.001

Positive	60	304	364			

Total	85	326	411			


**Table 7:** Relative frequency (prevalence) of two definitions of serum vitamin D deficiency before and after treatment with vitamin D3 injection.

vitamin D3 injection (Total	Before treatment		95% Confidence	After treatment		95% Confidence

examined N = 454)	N	%	Interval	N	%	Interval

Severe vitamin D deficiency (serum conc < 10 ng/ml)	119	26.2	(22.3 to 30.4)	17	3.7	(2.3 to 5.8)

vitamin D deficiency (serum conc < 20 ng/ml)	378	83.3	(79.6 to 86.5)	187	41.2	(36.7 to 45.8)


**Table 8:** Risk (paired odds ratio) of responding to vitamin D3 injection replacement therapy considering severe serum vitamin D deficiency as the outcome compared to pretreatment deficiency status.

	Sever vitamin D deficiency (serum conc < 10 ng/ml)-After treatment (within 12 weeks)			Paired	95% Confidence Interval	

vitamin D3 injection	Negative	Positive	Total	OR	OR	P

Severe vitamin D deficiency (serum conc < 10 ng/ml)-Baseline (before treatment)						

Negative	331	4	335	27	(10 to 73)	< 0.001

Positive	106	13	119			

Total	437	17	454			


**Table 9:** Risk (paired odds ratio) of responding to vitamin D3 injection replacement therapy considering vitamin D deficiency as the outcome compared to pretreatment deficiency status.

	vitamin D deficiency (serum conc < 20 ng/ml)-After treatment (within 12 weeks)			Paired	95% Confidence Interval	

vitamin D3 injection	Negative	Positive	Total	OR	Or	P

vitamin D deficiency (serum conc < 20 ng/ml)-Baseline (before treatment)						

Negative	57	19	76	11	(7 to 18)	< 0.001

Positive	210	168	378			

Total	267	187	454			


**Table 10:** Relative frequency (prevalence) of two definitions of serum vitamin D deficiency before and after treatment with a vitamin D2 injection + a D2 tablet.

vitamin D2 injection + D2 tablet	Before treatment		95% Confidence	After treatment		95% Confidence

(Total examined N = 1585)	N	%	Interval	N	%	Interval

Severe vitamin D deficiency (serum conc < 10 ng/ml)	622	39.2	(36.9 to 41.7)	72	4.5	(3.6 to 5.7)

vitamin D deficiency (serum conc < 20 ng/ml)	1462	92.2	(90.8 to 93.5)	1172	73.9	(71.7 to 76.1)


**Table 11:** Risk (paired odds ratio) of responding to vitamin D2 injection + D2 tablet replacement therapy considering severe serum vitamin D deficiency as the outcome compared to pretreatment deficiency status.

vitamin D2 injection + D2	Sever vitamin D deficiency (serum conc < 10 ng/ml)-After treatment (within 12 weeks)			Paired	95% Confidence Interval	

tablet	Negative	Positive	Total	OR	OR	P

Severe vitamin D deficiency (serum conc < 10 ng/ml)-Baseline (before treatment)						

Negative	941	22	963	26	(17 to 40)	< 0.001

Positive	572	50	622			

Total	1513	72	1585			


**Table 12:** Risk (paired odds ratio) of responding to vitamin D2 injection + D2 tablet replacement therapy considering severe serum vitamin D deficiency as the outcome compared to pretreatment deficiency status.

	vitamin D deficiency (serum conc < 20 ng/ml)-After treatment (within 12 weeks)			Paired	95% Confidence Interval	

vitamin D2 injection + D2 tablet	Negative	Positive	Total	OR	OR	P

vitamin D deficiency (serum conc < 20 ng/ml)-Baseline (before treatment)						

Negative	76	47	123	7	(5 to 10)	< 0.001

Positive	337	1125	1462			

Total	413	1172	1585			


**Table 13:** Relative frequency (prevalence) of two definitions of serum vitamin D deficiency before and after treatment with a vitamin D3 injection + a D2 tablet.

vitamin D3 injection + D2 tablet	Before treatment		95% Confidence	After treatment		95% Confidence

(Total examined N = 997)	N	%	Interval	N	%	Interval

Severe vitamin D deficiency (serum conc < 10 ng/ml)	314	31.5	(28.7 to 34.4)	14	1.4	(0.8 to 2.3)

vitamin D deficiency (serum conc < 20 ng/ml)	872	87.5	(85.3 to 89.4)	442	44.3	(41.3 to 47.4)


**Table 14:** Risk (paired odds ratio) of responding to vitamin D3 injection + D2 tablet replacement therapy considering severe serum vitamin D deficiency as the outcome compared to pretreatment deficiency status.

vitamin D3 injection	Sever vitamin D deficiency (serum conc < 10 ng/ml)-After treatment (within 12 weeks)			Paired	95% Confidence Interval	

+ D2 tablet	Negative	Positive	Total	OR	OR	P

Severe vitamin D deficiency (serum conc < 10 ng/ml)-Baseline (before treatment)						

Negative	679	4	683	76	(28 to 204)	< 0.001

Positive	304	10	314			

Total	983	14	997			


**Table 15:** Risk (paired odds ratio) of responding to vitamin D3 injection + D2 tablet replacement therapy considering severe serum vitamin D deficiency as the outcome compared to pretreatment deficiency status.

vitamin D3 injection	vitamin D deficiency (serum conc < 20 ng/ml)-After treatment (within 12 weeks)			Paired	95% Confidence Interval	

+ D2 tablet	Negative	Positive	Total	OR	OR	P

vitamin D deficiency (serum conc < 20 ng/ml)-Baseline (before treatment)						

Negative	103	22	125	21	(14 to 32)	< 0.001

Positive	452	420	872			

Total	555	442	997			

